# Finasteride Use: Evaluation of Depression and Suicide Risk

**DOI:** 10.1111/jocd.70102

**Published:** 2025-03-13

**Authors:** Aditya K. Gupta, Mary A. Bamimore, Greg Williams, Mesbah Talukder

**Affiliations:** ^1^ Mediprobe Research Inc. London Ontario Canada; ^2^ Division of Dermatology, Department of Medicine, Temerty Faculty of Medicine University of Toronto School of Medicine Toronto Ontario Canada; ^3^ Farjo Hair Institute London UK; ^4^ School of Pharmacy BRAC University Dhaka Bangladesh

**Keywords:** androgenetic alopecia, depression, finasteride use, suicide risk

## Abstract

**Background:**

Oral finasteride 1 mg/day is indicated for androgenetic alopecia (AGA), while 5 mg/day is for benign prostatic hyperplasia (BPH). Oral finasteride has been linked with depression and suicide; however, a causal association is uncertain. The so‐called post‐finasteride syndrome (PFS) refers to a “cluster” of side effects experienced by some men (i.e., cis men) after taking oral finasteride.

**Aims:**

The objective of the current study was to evaluate the association of depression and suicide with oral finasteride in males, using data from the United States Food and Drug Administration Adverse Event Reporting System (FAERS). As a secondary objective, we conducted disproportionality analyses of FAERS data to assess whether oral dutasteride use was linked to psychological symptoms related to depression and suicidality.

**Methods:**

We conducted disproportionality analyses for 5 AEs using MedDRA terms. Associations were metricized with the reporting odds ratio (ROR) across 3 time periods, namely, 2006–2011, 2013–2018, and 2019–2023.

**Results:**

No significant AEs/signals were detected with oral finasteride from 2006 to 2011 for any of the 5 AEs (completed suicide, depression suicidal, suicidal behavior, suicidal ideation, attempted suicide). Signals were detected for some AEs during 2013–2018 and 2019–2023. For example, there was a greater likelihood of reporting suicidal ideations in individuals taking oral finasteride during 2013–2018 (ROR = 2.8, *p* < 0.05) and 2019–2023 (ROR = 5.0, *p* < 0.05). In contrast, no signals were detected with oral dutasteride during 2006–2011, 2013–2018, and 2019–2023.

**Conclusion:**

The study found no significant correlation between oral finasteride and depression/suicide reports from 2006 to 2011 but noted a significant number of such reports in 2013–2018 and 2019–2023. This increase may be linked to heightened awareness of AEs following the recognition of so‐called PFS in 2012.

## 1 Introduction

Finasteride, a Type 2 5‐alpha reductase inhibitor (5‐ARI), is an oral therapeutic agent for androgenetic alopecia (AGA) [1], the most common form of hair loss in men. This 5‐ARI was initially approved in 1992 by the United States Food and Drug Administration (FDA) to treat benign prostatic hyperplasia (BPH) at a dose of 5 mg per day. In 1998, oral finasteride 1 mg per day was FDA‐approved to treat male AGA [1]. Clinical trials have investigated the effect of the 5 mg/day and 1 mg/day doses of oral finasteride on AGA in males and females; this study evaluates data in males only [2]. In clinical practice, oral finasteride 1 mg/day is preferred over 5 mg/day to treat male AGA.

As of 2012, reports have been emerging on the “post‐finasteride syndrome” [3], a term that alleges the use of oral finasteride 1 mg/day to be linked to a cluster of conditions that impact psychological and/or sexual health [3, 4, 5, 6, 7, 8].

The United States Food and Drug Administration Adverse Event Reporting System (FAERS) started being a repository for spontaneously reported AE data in 1969 [9, 10, 11]. However, the structure of this database was majorly reformed in 1998 [9, 10, 11]. This repository currently has freely accessible data from 2004 onwards. Though awareness of post‐finasteride syndrome (PFS) is marked in and out of the peer‐reviewed literature, the International Classification of Diseases (ICD) currently does not recognize it as a condition. Nonetheless, the aim of the current study was to determine whether psychological symptoms associated with the “so‐called” PFS (i.e., depression and suicide) can be detected as significantly reported AEs (i.e., signals) in the FAERS database.

## 2 Methods

### 2.1 Data Source

The conduct of the present study was in accordance with the *Strengthening the Reporting of Observational Studies in Epidemiology* (STROBE) guidelines [12]. We analyzed data from FAERS to determine if oral finasteride use is significantly associated with AEs related to depression and suicide. The FAERS is a database that constitutes data from seven files (i.e., the “DEMO,” “DRUG,” “INDI,” “OUTC,” “REAC,” “RPSR” and “THER” datasets) that, since 1969 [13], are published on a quarterly basis. To determine whether a signal could be detected for each AE, we first mined data from 3 of the 7 FAERS files (i.e., the “DEMO,” “DRUG” and “REAC” files datasets) and then conducted a case/non‐case disproportionality analysis.

### 2.2 Outcomes

We used the literature [14, 15] to inform our data mining which involved appending, merging and deduplicating the data files. The 5 AEs, as per the *Medical Dictionary for Regulatory Activities* (MedDRA) nomenclature (version 27.0), were “completed suicide,” “depression suicidal,” “suicidal behavior,” “suicidal ideation,” and “suicide attempt.” For the disproportionality analysis that was conducted for each AE, we used van Puijenbroek et al.'s [16] formula for the reporting odds ratio (ROR) and its corresponding 95% confidence interval (CI); we chose the ROR because it is not only the most straightforward metric for signal detection but is also very commonly used [17]. ROR can be interpreted as the likelihood of reporting an AE when the drug of interest (i.e., oral finasteride herein) is also reported, in reference to the likelihood of reporting an AE when the drug of interest is not reported.

### 2.3 Reporting Odds Ratio

Below is the formula for ROR estimation, as per van Puijenbroek et al.'s [16]:

**Table:** 

Contingency table for count data used for the computation of a ROR and its 95% CI			1.1
	Intervention of interest	Other interventions	
Adverse event of interest	*a*	*b*	
Other adverse event	*c*	*d*	
Reporting odds ratio (ROR) = $\frac{a/c}{b/d}$			1.2
Lower bound of 95% confidence interval for ROR = $e^{\ln\left(\mathrm{ROR}\right)-1.96\;\sqrt{\frac{1}{a}+\frac{1}{b}+\frac{1}{c}+\frac{1}{d}}}$			1.3
Upper bound of 95% confidence interval for ROR = $e^{\ln\left(\mathrm{ROR}\right)+1.96\;\sqrt{\frac{1}{a}+\frac{1}{b}+\frac{1}{c}+\frac{1}{d}}}$			1.4
(For Equations 1.3 and 1.4, ROR, *a*, *b*, *c* and *d* correspond to those in 1.1 and 1.2)			

Estimating the ROR and its corresponding 95% CI begins with obtaining counts of the following: (i) reports with the intervention of interest and adverse event of interest (i.e., “a” in 1.1), (ii) reports with all other interventions and adverse event of interest (i.e., “b” in 1.1), (iii) reports with the intervention of interest and all other adverse events (i.e., “c” in 1.1), and (iv) reports with all other interventions and all other adverse events (i.e., “d” in 1.1). Once these counts (i.e., “a,” “b,” “c,” and “d”) are obtained, the formulae in 1.2 to 1.4 were used to estimate the ROR and 95% CI.

A signal is detected when the ROR is statistically significant (*p* < 0.05); the 95% CI of a statistically significant ROR does not have the null value, which simply means that the value of the lower bound of the 95% CI is greater than 1. So, if the ROR for an AE is, for example, 1.20 but the lower bound of the 95% CI is 0.80, this AE did not produce a signal. However, if the same AE with a ROR of 1.20 had a 95% CI of 1.18 to 2.1, this AE has produced a signal because 1.18 (i.e., the lower bound of the 95% CI) is greater than 1.

The disproportionality analyses were conducted for each of the 5 AEs for any dose (no restriction by dose throughout data mining), 1 and 5 mg. Furthermore, the analyses were also conducted across 3 “eras” (2006–2011), (2013–2018) (2019–2023) for these 3 dose groups (1 and 5 mg, no dose restriction [i.e., any dose]). The first and second eras corresponded to 6 years before and after the first publicizing of the so‐called post‐finasteride syndrome, that is, 2006 to 2011 (inclusive) and 2013 to 2018 (inclusive); the third era corresponded to 2019 to 2023 (inclusive). We excluded FAERS data from 2012. The year 2012 marks a significant point in oral finasteride's history since awareness of the so‐called PFS “formally” commenced in 2012 by the Post Finasteride Syndrome Foundation. We excluded 2012 data from our analyses as it was the year the PFS Foundation “formalized” awareness of the so‐called PFS.

According to CDC data, suicide rates generally increase with age, with the highest rates observed among older adults, particularly men. The highest suicide rate was observed among individuals aged 75 and older, at 20.3 per 100 000. Among youth and young adults aged 10 to 24, the suicide rate was 11.0 per 100 000 [18]. We performed regression analyses (multivariable logistic regression) to determine whether the association between the respective AE and finasteride use was confounded by age. Each regression analysis corresponded to the following equation.

$${\hat{Y}}_{\mathrm{AE}}={\hat{\beta }}_{\text{intervention}}+{\hat{\beta }}_{\mathrm{age}}+{\hat{\beta }}_{0}$$

where, $\hat{\beta }$_age_, $\hat{\beta }$_intervention_, and $\hat{\beta }$_0_ corresponds to the expected regression coefficient for age (as a continuous variable), intervention (the respective dose group of finasteride vs. all other drugs), and the intercept; $\hat{Y}$_AE_ corresponds to the occurrence of the adverse event of interest (as a binary variable).

As a secondary aim, we also determined, through disproportionality analyses of FAERS data, whether oral dutasteride (which is a Type 1 and Type 2 5‐ARI) use was associated with psychological symptoms associated with the “so‐called” PFS.

**FIGURE 1:**
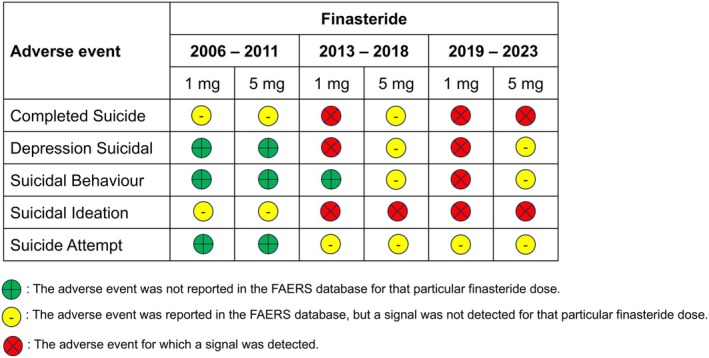
Association between oral finasteride use and psychological impact from 2006 to 2011, 2013 to 2018, and 2019 to 2023.

**FIGURE 2:**
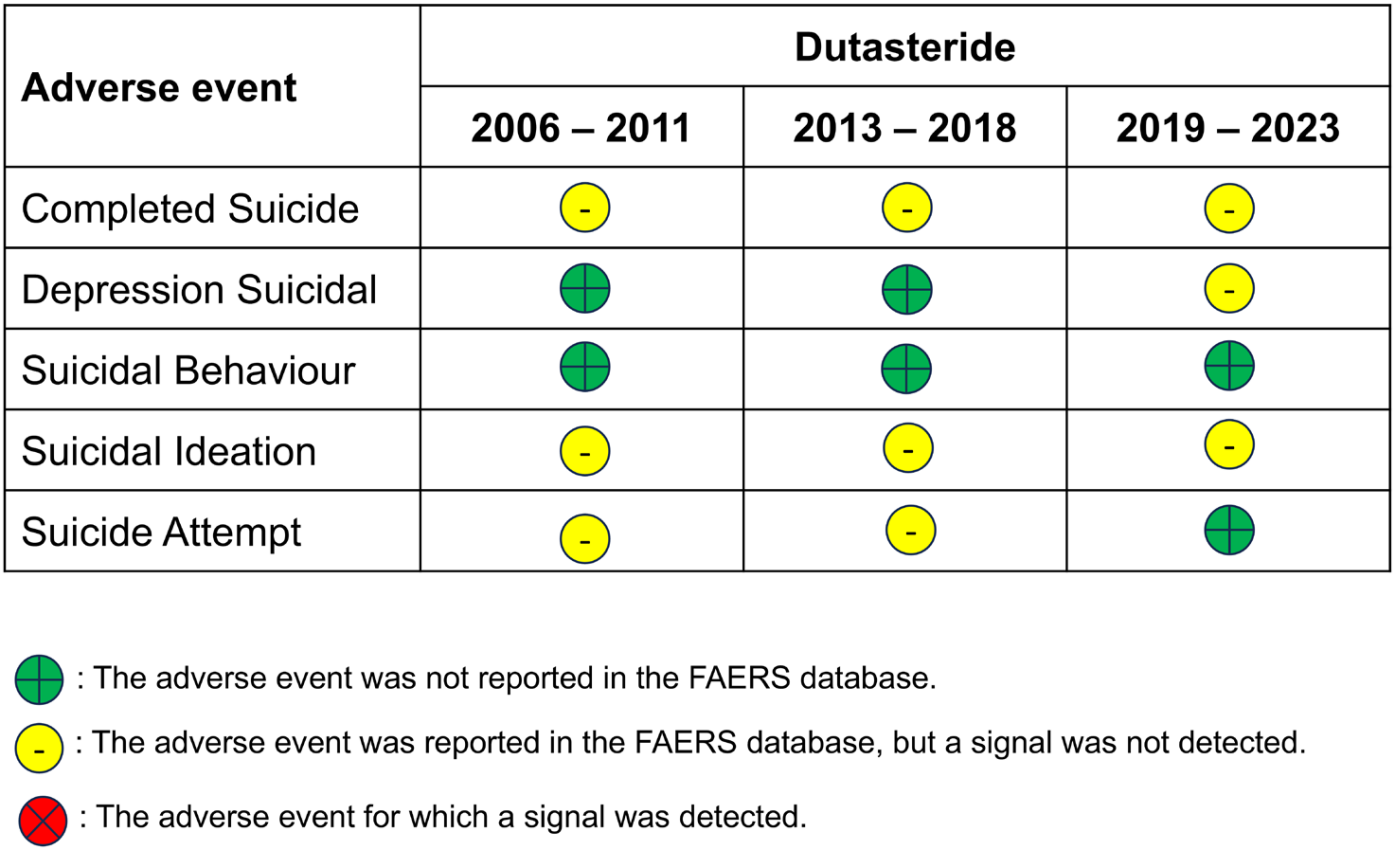
Association between oral dutasteride use and psychological impact from 2006 to 2011, 2013 to 2018, and 2019 to 2023.

We created figures for the two 5‐ARIs, where we—qualitatively—depicted the RORs with different colors: yellow (AE was reported but no signal detected), green (AE was not reported) and red (AE yielded a signal) (Figures 1 and 2).

## 3 Results

Our findings pertaining to disproportionality analyses for any dose, 1 and 5 mg of oral finasteride across the 6‐year period a year before the “formal” commencement of the awareness of the so‐called PFS (i.e., a year before “PFS awareness”) are summarized in Table 1; herein this paper, we describe this period as “pre‐PFS era (2006–2011).” Results for the 6‐year period after the so‐called PFS awareness are presented in Table 2; we refer to this timeframe as “1st post‐PFS era (2013–2018).” Table 3 details our findings for 5‐year period seven years after PFS awareness—and this observation period we denote as “2nd post‐PFS era (2019–2023).”

**TABLE 1:** Association between oral finasteride use and psychological impact prior to the publicizing of the “post‐finasteride syndrome” from 2006 to 2011 (inclusive) across any dose, 1 and 5 mg.

Adverse event	2006–2011 (inclusive), *N* = 31 724 432 (any dose)			2006–2011 (inclusive), *N* = 198 612 (1 mg)			2006–2011 (inclusive), *N* = 1 311 037 (5 mg)			*p*‐value for Breslow‐Day test for homogeneity
	ROR	Lower bound of 95% CI	Upper bound of 95% CI	ROR	Lower bound of 95% CI	Upper bound of 95% CI	ROR	Lower bound of 95% CI	Upper bound of 95% CI	
Completed suicide	0.266	0.143	0.494	0.805	0.113	5.743	0.525	0.074	3.728	0.761
Depression suicidal	—	—	—	—	—	—	—	—	—	—
Suicidal behavior	0.423	0.06	2.999	—	—	—	—	—	—	—
Suicidal ideation	1.143	0.866	1.509	1.772	0.976	3.218	0.627	0.235	1.672	0.064
Suicide attempt	0.198	0.089	0.439	—	—	—	—	—	—	—

*Note:* Between 2006 and 2011 there were no statistically significant AEs (signals) reported. *p*‐values from the Breslow‐Day test for homogeneity across the two groups of doses (i.e., the 1 and 5 mg groups) are presented in the rightmost column.

Abbreviations: CI, confidence interval; ROR, reporting odds ratio.

**TABLE 2:** Association between oral finasteride use and psychological impact after the publicizing of the “post‐finasteride syndrome” from 2013 to 2018 (inclusive) across any dose, 1 and 5 mg.

Adverse event	2013–2018 (inclusive), *N* = 64 659 416 (any dose)			2013–2018 (inclusive), *N* = 645 855 (1 mg)			2013–2018 (inclusive), *N* = 3 538 440 (5 mg)			*p*‐value for Breslow‐Day test for homogeneity
	ROR	Lower bound of 95% CI	Upper bound of 95% CI	ROR	Lower bound of 95% CI	Upper bound of 95% CI	ROR	Lower bound of 95% CI	Upper bound of 95% CI	
Completed suicide	0.96	0.779	1.183	**3.963**	**2.62**	**5.994**	0.733	0.329	1.634	< 0.001
Depression suicidal	**4.614**	**2.903**	**7.335**	**17.849**	**9.2**	**34.627**	2.163	0.535	8.753	0.003
Suicidal behavior	1.247	0.594	2.617	—	—	—	0.543	0.077	3.872	—
Suicidal ideation	**2.827**	**2.538**	**3.148**	**3.628**	**3.031**	**4.341**	**1.847**	**1.453**	**2.347**	< 0.001
Suicide attempt	0.663	0.498	0.883	0.955	0.611	1.493	0.216	0.09	0.52	0.001

*Note:* Gray colored cells with bold text represent statistically significant (*p <* 0.05) association which correspond to a detected signal. *p*‐values Breslow‐Day test for homogeneity across the two groups of doses (i.e., the 1 and 5 mg groups) are presented in the right most column.

Abbreviations: CI, confidence interval; ROR, reporting odds ratio.

**TABLE 3:** Association between oral finasteride use and psychological impact after the publicizing of the “post‐finasteride syndrome” from 2019 to 2023 (inclusive) across any dose, 1 and 5 mg.

Adverse event	2019–2023 (inclusive), *N* = 70 221 981 (any dose)			2019–2023 (inclusive), *N* = 296 569 (1 mg)			2019–2023 (inclusive), *N* = 1 103 415 (5 mg)			*p*‐value for Breslow‐Day test for homogeneity
	ROR	Lower bound of 95% CI	Upper bound of 95% CI	ROR	Lower bound of 95% CI	Upper bound of 95% CI	ROR	Lower bound of 95% CI	Upper bound of 95% CI	
Completed suicide	**1.705**	**1.353**	**2.149**	**8.967**	**5.611**	**14.3**	**2.354**	**1.044**	**5.312**	0.03
Depression suicidal	**8.806**	**6.184**	**12.541**	**49.567**	**24.495**	**100.303**	3.368	0.815	13.924	< 0.001
Suicidal behavior	**3.225**	**1.784**	**5.828**	**6.625**	**1.976**	**22.216**	1.212	0.168	8.747	0.113
Suicidal ideation	**4.956**	**4.466**	**5.501**	**9.893**	**8.027**	**12.193**	**2.37**	**1.7**	**3.305**	< 0.001
Suicide attempt	0.709	0.502	1.003	1.622	0.863	3.049	0.841	0.399	1.771	0.180

*Note:* Gray colored cells with bold text represent statistically significant (*p <* 0.05) association which correspond to a detected signal. *p*‐values Breslow‐Day test for homogeneity across the two groups of doses (i.e., the 1 and 5 mg groups) are presented in the right most column.

Abbreviations: CI, confidence interval; ROR, reporting odds ratio.

No signal was detected for any of the 5 neuropsychiatric AEs throughout the pre‐PFS era (2006–2011) (Table 1). The absolute frequencies of each of the 5 AEs are presented in the Supporting Information.

Figure 1 summarizes the association between oral finasteride use and psychological impact during the “pre‐PFS era (2006–2011)” the “1st post‐PFS era (2013–2018)” and the “2nd post‐PFS era (2019–2023).”

### 3.1 Suicide Attempt

Across all three eras, no signal was detected for suicide attempts (Tables 1, 2, 3).

### 3.2 Completed Suicide

Prior to 2011, that is, in the Pre‐PFS era (2006–2011), there was no signal for completed suicide. Regarding completed suicide in the 1st post‐PFS era (2013–2018), a signal was detected for 1 mg oral finasteride (ROR = 3.96, 95% CI: 2.62–5.99) (Table 2); in the 2nd post‐PFS era (2019–2023), a signal was detected for oral finasteride in any dose (ROR = 1.705, 95% CI: 1.35–2.15), 1 mg (ROR = 8.97, 95% CI: 5.61–14.3) and 5 mg (ROR = 2.35, 95% CI: 1.04–5.31) (Table 3). Results from the Breslow‐Day test for homogeneity showed that the detected signal at the 1 mg dose was significantly (*p* < 0.05) stronger than that at the 5 mg dose (Table 3) in the 2nd post‐PFS era (2019–2023).

Results from our logistic regression support that, across the 1st post‐PFS era (2013–2018), there is an association between the reporting of this AE and 1 mg of oral finasteride even after controlling for confounding due to age (Table 4).

**TABLE 4:** Association between dutasteride use and psychological impact before and after the publicizing of the “post‐finasteride syndrome.”

Adverse event	2006–2011 (inclusive), *N* = 31 724 432			2013–2018 (inclusive), *N* = 64 659 416			2019–2023 (inclusive), *N* = 70 221 981		
	ROR	Lower bound of 95% CI	Upper bound of 95% CI	ROR	Lower bound of 95% CI	Upper bound of 95% CI	ROR	Lower bound of 95% CI	Upper bound of 95% CI
Completed suicide	1.724	0.647	4.600	0.275	0.124	0.611	0.163	0.041	0.652
Depression suicidal	—	—	—	—	—	—	2.918	0.941	9.051
Suicidal behavior	—	—	—	—	—	—	—	—	—
Suicidal ideation	0.369	0.052	2.622	0.495	0.293	0.835	0.57	0.324	1.003
Suicide attempt	0.532	0.075	3.779	0.592	0.319	1.101	—	—	—

*Note:* Of all the RORs that were estimated and presented in this table, in the periods 2006–2011, 2013–2018, and 2019–2023 no signals were detected because the lower bound of all RORs are below 1.00.

Abbreviations: CI, confidence interval; ROR, reporting odds ratio.

### 3.3 Depression Suicidal

In the pre‐PFS era (2006–2011), there was no signal for depression suicidal for any dose of oral finasteride.

During the 1st post‐PFS era (2013–2018), a signal was detected for this AE with the use of oral finasteride at any dose (ROR = 4.61, 95% CI: 2.90–7.34) and 1 mg (ROR = 17.85, 95% CI: 9.20–34.63); no signal was detected for this AE at the 5 mg dose across this era (Table 2).

Similarly, in the 2nd post‐PFS era (2019–2023), this AE was significantly associated with the use of oral finasteride at any dose (ROR = 8.81, 95% CI: 6.18–12.54) and 1 mg (ROR = 49.57, 95% CI: 24.50–100.3) (Table 3).

Through our regression analyses, we found that there was a statistically significant association between this AE and the use of oral finasteride at any dose and 1 mg, even after adjusting for age. However, the multivariable model showed no significant association between this outcome and the 1 mg dose of this agent. Hence, our multivariable regression supports that the signal detected for depression suicidal with 1 mg finasteride is likely to have been confounded by variation in age (Table 4).

### 3.4 Suicidal Behavior

In the pre‐PFS era (2006–2011), there was no signal for suicidal behavior for any dose of finasteride.

A signal was detected for this AE only in the 2nd post‐PFS era (2019–2023); furthermore, a signal was detected for any dose (ROR = 3.23, 95% CI: 1.78–5.83) and 1 mg (ROR = 6.63, 95% CI: 1.98–22.22) of oral finasteride (Table 3). Results of our regression showed that, after adjusting for confounding due to age, the AE was still significantly associated with the use of oral finasteride at 1 mg and any dose (Table 4).

### 3.5 Suicidal Ideation

In the pre‐PFS era (2006–2011) there was no signal for suicidal ideation for any dose of oral finasteride.

Across the 1st post‐PFS era (2013–2018), a signal was detected for suicidal ideation with oral finasteride of any dose (ROR = 2.83, 95% CI: 2.54–3.15), 1 mg (ROR = 3.63, 95% CI: 3.03–4.34) and 5 mg (ROR = 1.85, 95% CI: 1.45–2.35) (Table 2).

Likewise, in the 2nd post‐PFS era (2019–2023), a signal was detected with the use of oral finasteride at any dose (ROR = 4.96, 95% CI: 4.47–5.50), 1 mg (ROR = 9.90, 95% CI: 8.03–12.19) and 5 mg (ROR = 2.37, 95% CI: 1.70–3.31) (Table 3).

### 3.6 Dutasteride

Results from our disproportionality analyses for oral dutasteride showed no signal before nor after the publicizing of the so‐called PFS (Table 4, Figure 2). This is why Figure 2, unlike Figure 1, has no red circles (because they were no signals significant AEs).

## 4 Discussion

Multiple clinical studies suggest that oral finasteride treatment may increase the risk of neuropsychiatric side effects. A systematic review found that the odds of developing depressive symptoms were significantly higher in oral finasteride users compared to non‐users, with an odds ratio of 2.14 [19]. The pooled rates of depressive symptoms were higher with oral finasteride at 3.33% compared to 2.54% without it. Additionally, the risk of suicidal ideation or behavior was greater with oral finasteride at 21.2% versus 14.0% without it (*p* < 0.0001) [19]. Besides, reports have noted that nearly 50% of men experiencing so‐called post‐finasteride syndrome reported clinically significant depression [20].

In a recent study, Laanani et al. [20] reported that males treated for BPH with finasteride demonstrated no increased risk of suicidal behavior compared to those on dutasteride therapy, especially among males without psychiatric disorders. However, in males with a history of mood disorders, finasteride may be associated with a higher risk of suicide death or severe self‐harm [20]. It is important to note that several of these studies investigating the mental health impacts of finasteride lacked appropriate control groups, which limits our ability to draw definitive conclusions about causality.

In this study, we investigated whether reporting in FAERS database could detect a signal (significant AE) for AEs as per 5 MedDRA preferred terms, namely, “completed suicide,” “depression suicidal,” “suicidal behavior,” “suicidal ideation” and “suicide attempt”. Though oral finasteride has been implicated in the effecting of neuropsychiatric AEs, a causal link has not been established, as pointed out in the systematic review by Hirshburg et al. [21].

In the era prior to 2012 (2006–2011), no signal was detected in the FAERS database for any of the 5 neuropsychiatric AEs. However, in the two eras spanning 2013 to 2023, we found signals for oral finasteride. This finding is congruent with that of Nguyen et al. [6] who also found no signal for neuropsychiatric AEs before 2012 but did find one thereafter. Like Nguyen et al. [6] we, by and large, also captured more cases with the finasteride 1 mg/day than with the 5 mg/day dose. Notably, during the 2nd post‐PFS eras, the highest number of signals linked with finasteride 1 mg/day (signals for completed suicide, depression‐related suicidality, suicidal behavior, and suicidal ideation) may have been influenced by the COVID‐19 pandemic as a confounding factor.

Overall, our findings show that reports of finasteride‐related neuropsychiatric side effects have been more frequent in the FAERS database after 2012. This increase may be due to several factors: in 2012, the US FDA updated the finasteride package insert to include information on the so‐called PFS; an article on persistent sexual dysfunction and depressive symptoms associated with oral finasteride gained significant media attention around that time; and in July 2012, the PFS Foundation was established to support research on so‐called PFS [4, 5, 6, 7].

In comparison, oral dutasteride (Type 1 and Type 2 5‐ARI) showed no signal before nor after the publicizing of the so‐called PFS in 2012, which supports the possibility that the signals we detected for oral finasteride could be attributed to a “disproportion” in reporting (i.e., reporting bias—or the selection bias due to over‐reporting).

Notably, pharmacovigilance studies using the FAERS database have limitations such as incomplete, inconsistent, and duplicate reports, as well as significant under‐reporting of AEs. While the database is useful for identifying safety signals, the findings require cautious interpretation and further validation.

## 5 Conclusion

Our study found no significant link between oral finasteride and reports of depression or suicide from 2006 to 2011. However, a significant number of such reports emerged during 2013–2018 and 2019–2023, potentially due to increased awareness of adverse effects following the identification of PFS in 2012. However, to ensure patient safety, all patients should be screened for mood alterations, depression, and suicidal ideation by a health care professional prior to being prescribed finasteride [22]. All patients receiving oral finasteride should be advised to discontinue the medication and seek medical advice if they feel depressed, suicidal, or experience mood alterations [22, 23]. Patients on oral finasteride should be monitored regularly by a health care professional for adverse effects. Males with a history of mood disorders may not be suitable candidates to receive finasteride or may need regular, closely supervised follow‐up by a physician with specialist training in psychiatry.

## Author Contributions

Conception of the manuscript was done by A.K.G. Data analysis was performed by M.A.B. The manuscript was drafted by A.K.G., M.T., G.W., and M.A.B.; it was substantively edited and revised by A.K.G., M.T., G.W., and M.A.B.

## Ethics Statement

The authors have nothing to report.

## Conflicts of Interest

The authors declare no conflicts of interest.

## Supporting information


Tables S1–S4.

## Data Availability

The data that support the findings of this study are available from the corresponding author upon reasonable request.
