# Supplementary material for: Finasteride Use: Evaluation of Depression and Suicide Risk
Source: J Cosmet Dermatol. 2025 Mar 13;24(3):e70102. doi: 10.1111/jocd.70102 (PMC11906302; doi:10.1111/jocd.70102)
Supplement: Supplementary file 1 — Tables S1–S4. [file JOCD-24-e70102-s001.docx]

**Supplementary Information**

**Table S1.** Occurrence of psychology-related adverse events from using (vs. not using) finasteride (in any dose, 1mg and 5mg) between 2006 to 2011 (inclusive)

| AE | Any dose of finasteride  vs. all other drugs  (%)  n = 31,724,432 | | 1mg of finasteride  vs. all other drugs  (%)  n = 198,612 | | 5mg of finasteride  vs. all other drugs  (%)  n = 1,311,037 | |
| --- | --- | --- | --- | --- | --- | --- |
| Completed suicide vs. all other AEs | 0.05 | 0.17 | 0.09 | 0.11 | 0.04 | 0.07 |
| Depression suicidal vs. all other AEs | - | - | - | - | - | - |
| Suicidal behavior vs. all other AEs | 0.01 | 0.02 | - | - | - | - |
| Suicidal ideation vs. all other AEs | 0.22 | 0.19 | 0.91 | 0.51 | 0.14 | 0.22 |
| Suicide attempt vs. all other AEs | 0.03 | 0.13 | - | - | - | - |

**Abbreviations:**

AE = adverse event,

mg = milligram,

n = number of observations (or sample size),

**Notes:**

- The information in this table was based on the United States Food and Drug Administration Adverse Event Reporting System (FAERS) database. Herein, we presented a descriptive summary for the relative occurrence of 5 psychology-related AEs previously reported to be associated with use of finasteride.
- To understand the content of this table, below is an explanation for the first row:
- Across reports pertaining to any dose of finasteride, the occurrence completed suicide—relative to all other adverse events—was 0.05% across 2006 to 2011 (inclusive); across reports pertaining to all other drugs, the occurrence of completed suicide—relative to all other adverse events—was 0.17% across 2006 to 2011 (inclusive)

**Table S2.** Occurrence of psychology-related adverse events from using (vs. not using) oral finasteride (in any dose, 1mg and 5mg) between 2013 to 2018 (inclusive)

| AE | Any dose of finasteride  vs. all other drugs  (%)  n = 64,659,416 | | 1mg of finasteride  vs. all other drugs  (%)  n = 645,855 | | 5mg of finasteride  vs. all other drugs  (%)  n = 3,538,440 | |
| --- | --- | --- | --- | --- | --- | --- |
| Completed suicide vs. all other AEs | 0.1 | 0.1 | 0.11 | 0.03 | 0.03 | 0.03 |
| Depression suicidal vs. all other AEs | 0.02 | 0.01 | 0.07 | 0.01 | 0.01 | 0.01 |
| Suicidal behavior vs. all other AEs | 0.01 | 0.01 | - | - | - | - |
| Suicidal ideation vs. all other AEs | 0.36 | 0.13 | 0.56 | 0.16 | 0.24 | 0.13 |
| Suicide attempt vs. all other AEs | 0.06 | 0.08 | 0.09 | 0.09 | 0.02 | 0.09 |

**Abbreviations:**

AE = adverse event,

mg = milligram,

n = number of observations (or sample size),

**Notes:**

- The information in this table was based on the United States Food and Drug Administration Adverse Event Reporting System (FAERS) database. Herein, we presented a descriptive summary for the relative occurrence of 5 psychology-related AEs previously reported to be associated with use of finasteride.
- To understand the content of this table, below is an explanation for the first row:
- Across reports pertaining to any dose of oral finasteride, the occurrence completed suicide—relative to all other adverse events—was 0.1% across 2013 to 2018 (inclusive); across reports pertaining to all other drugs, the occurrence of completed suicide—relative to all other adverse events—was 0.1% across 2013 to 2018 (inclusive)

**Table S3.** Occurrence of psychology-related adverse events from using (vs. not using) finasteride (in any dose, 1mg and 5mg) between 2019 to 2023 (inclusive)

| AE | Any dose of finasteride  vs. all other drugs  (%)  n = 70,221,981 | | 1mg of finasteride  vs. all other drugs  (%)  n = 296,569 | | 5mg of finasteride  vs. all other drugs  (%)  n = 1,103,415 | |
| --- | --- | --- | --- | --- | --- | --- |
| Completed suicide vs. all other AEs | 0.11 | 0.07 | 0.34 | 0.04 | 0.04 | 0.02 |
| Depression suicidal vs. all other AEs | 0.05 | 0.01 | 0.26 | 0.01 | 0.02 | 0.01 |
| Suicidal behavior vs. all other AEs | 0.02 | 0.01 | 0.05 | 0.01 | 0.01 | 0.01 |
| Suicidal ideation vs. all other AEs | 0.54 | 0.11 | 1.76 | 0.18 | 0.23 | 0.1 |
| Suicide attempt vs. all other AEs | 0.05 | 0.07 | 0.16 | 0.1 | 0.05 | 0.06 |

**Abbreviations:**

AE = adverse event,

mg = milligram,

n = number of observations (or sample size)

**Notes:**

- The information in this table was based on the United States Food and Drug Administration Adverse Event Reporting System (FAERS) database. Herein, we presented a descriptive summary for the relative occurrence of 5 psychology-related AEs previously reported to be associated with use of finasteride.
- To understand the content of this table, below is an explanation for the first row:
- Across reports pertaining to any dose of finasteride, the occurrence completed suicide—relative to all other adverse events—was 0.11% across 2019 to 2023 (inclusive); across reports pertaining to all other drugs, the occurrence of completed suicide—relative to all other adverse events—was 0.07% across 2019 to 2023 (inclusive)

**Table S4. Regression analyses for association between the reporting of respective AEs and age.**

| **2018 – 2023** | | | | | | | | | | | | |
| --- | --- | --- | --- | --- | --- | --- | --- | --- | --- | --- | --- | --- |
| *Any dose* | | | | | | | | | | | | |
| Covariate | Simple regression | | | | | | Multivariable regression | | | | | |
|  | Depression Suicidal | | | Suicidal Ideation | | | Depression Suicidal | | | Suicidal Ideation | | |
| Age  (years) | -0.018*** | | | -0.027*** | | | -0.018*** | | | -0.027*** | | |
| Finasteride (any dose) vs. all other drugs |  | | |  | | | -1.685*** | | | -1.236*** | | |
| N | 52,067,174 | | | | | | 52,067,174 | | | | | |
|  |  | | | | | |  | | | | | |
| *1mg* | | | | | | | | | | | | |
| Covariate | Simple regression | | | | | | Multivariable regression | | | | | |
|  | Completed Suicide | | Depression Suicidal | | Suicidal Ideation | | Completed Suicide | | Depression Suicidal | | Suicidal Ideation | |
| Age  (years) | -0.023*** | | -0.018*** | | -0.027*** | | -0.023*** | | -0.018*** | | -0.027*** | |
| Finasteride 1mg  vs. all other drugs |  | |  | |  | | 1.274*** | | -0.323 | | -0.288*** | |
| N | 52,067,174 | | | | | | 52,067,174 | | | | | |
| *5mg* | | | | | | | | | | | | |
| Covariate | Simple regression | | | | | | Multivariable regression | | | | | |
|  | Suicidal Ideation | | | | | | Suicidal Ideation | | | | | |
| Age  (years) | -0.027*** | | | | | | -0.027*** | | | | | |
| Finasteride 5mg  vs. all other drugs |  | | | | | | -0.095*** | | | | | |
| N | 52,067,174 | | | | | | 52,067,174 | | | | | |
| **2019 – 2023** | | | | | | | | | | | | |
| *Any dose* | | | | | | | | | | | | |
| Covariate | Simple regression | | | | | | Multivariable regression | | | | | |
|  | Completed Suicide | Depression Suicidal | | Suicidal Behavior | | Suicidal Ideation | Completed Suicide | Depression Suicidal | | Suicidal Behaviour | | Suicidal Ideation |
| Age  (years) | -0.015*** | -0.002*** | | -0.002*** | | -0.027*** | -0.015*** | -0.002*** | | -0.002*** | | -0.027*** |
| Finasteride any dose  vs. all other drugs |  |  | |  | |  | 0.200 | 2.111*** | | 1.320*** | | 1.909*** |
| N | 53,340,771 | | | | | | 53,340,771 | | | | | |
| *1mg* | | | | | | | | | | | | |
| Covariate | Simple regression | | | | | | Multivariable regression | | | | | |
|  | Completed Suicide | Depression Suicidal | | Suicidal Behaviour | | Suicidal Ideation | Completed Suicide | Depression Suicidal | | Suicidal Behaviour | | Suicidal Ideation |
| Age  (years) | -0.002*** | -0.002*** | | -0.003*** | | -0.0001*** | -0.002*** | -0.002*** | | -0.0001*** | | -0.0001*** |
| Finasteride 1mg  vs. all other drugs |  |  | |  | |  | 0.541*** | -0.560** | | -0.581** | | -0.737*** |
| N | 53,340,905 | | | | | | 53,340,905 | | | | | |
| *5mg* | | | | | | | | | | | | |
| Covariate | Simple regression | | | | | | Multivariable regression | | | | | |
|  | Completed Suicide | | | Suicidal Ideation | | | Completed Suicide | | | Suicidal Ideation | | |
| Age  (years) | -0.015*** | | | -0.027*** | | | -0.015*** | | | -0.027*** | | |
| Finasteride 5mg  vs. all other drugs |  | | |  | | | 1.275*** | | | -0.093*** | | |
| N | 57,481,565 | | | 57,481,565 | | | 57,481,565 | | | 57,481,565 | | |
